# Ocular Manifestations in Pediatric Traumatic Brain Injury Admitted to the ICU: A Prospective Analysis

**DOI:** 10.3390/vision9040082

**Published:** 2025-10-04

**Authors:** Amer Jaradat, Rami Al-Dwairi, Adam Abdallah, Atef F. Hulliel, Rawhi Alshaykh, Mahmood Al Nuaimi, Ala’ Al Barbarawi, Seren Al Beiruti, Abdelwahab Aleshawi

**Affiliations:** 1 Department of Neurosurgery, Faculty of Medicine, Jordan University of Science & Technology, Irbid 22110, Jordan; dradamabdallah@gmail.com; 2 Department of Special Surgery, Division of Ophthalmology, Faculty of Medicine, Jordan University of Science & Technology, Irbid 22110, Jordan; mahmoud.alnuaimi93@gmail.com (M.A.N.); ala.barbarawi@yahoo.com (A.A.B.); seren.beiruti@yahoo.com (S.A.B.); abdelwahhabjamal@yahoo.com (A.A.); 3 Faculty of Medicine, Jordan University of Science & Technology, Irbid 22110, Jordan; afhulliel181@med.just.edu.jo (A.F.H.); rawhiyasin.me@gmail.com (R.A.)

**Keywords:** traumatic brain injury, ocular, pediatric, prospective analysis

## Abstract

Background: Traumatic Brain Injury (TBI) in children is a major cause of morbidity and mortality worldwide. Ocular manifestations are common but often overlooked, despite their potential to cause long-term visual impairment. This study aimed to evaluate the prevalence and characteristics of ocular findings in pediatric TBI patients admitted to the intensive care unit (ICU). Method: We prospectively reviewed records of pediatric patients (≤16 years) with TBI admitted to the Neurosurgery ICU at King Abdullah University Hospital (January 2022–December 2024). TBI was defined using U.S. CDC criteria and confirmed by clinical and radiological findings. Ocular manifestations were identified from ophthalmology consultations, neurosurgical notes, and bedside examinations. Demographics, injury details, and clinical outcomes were recorded. Statistical analyses included Chi-square, Fisher’s exact, and Mann–Whitney U tests, with significance set at *p* ≤ 0.05. Results: Thirty-eight patients (median age: 8 years; 55.3% male) were included. Ocular findings were present in 20 patients (52.6%). These patients were significantly older (median age 10 vs. 6 years, *p* = 0.007) and had lower admission GCS scores (11 vs. 14, *p* = 0.016). Male predominance was higher in the ocular group (75.0% vs. 33.3%, *p* = 0.030). Ocular findings were significantly associated with surgical intervention (60.0% vs. 22.2%, *p* = 0.025), orbital fractures (40.0% vs. 5.6%, *p* = 0.021), basal skull fracture signs (*p* = 0.036), and extraocular muscle limitation (*p* = 0.048). On multivariable analysis, orbital fracture remained the only independent predictor of ocular findings (aOR 2.22, 95% CI 1.17–3.57, *p* = 0.02). Conclusion: Over half of pediatric ICU TBI patients demonstrated ocular manifestations, closely linked to greater injury severity and craniofacial trauma. Routine, comprehensive ophthalmological evaluation should be integrated into the multidisciplinary management of severe pediatric TBI to optimize visual and functional outcomes.

## 1. Introduction

Traumatic Brain Injury (TBI) in the pediatric population represents a significant global health concern, leading to substantial morbidity and mortality. Children are particularly vulnerable to TBI due to their developing brains and unique anatomical characteristics, making the assessment and management of these injuries critical [1]. The worldwide incidence of pediatric TBI ranges broadly, typically reported between 47 and 280 per 100,000 children annually, affecting more than 3 million children globally each year [2]. In the United States alone, an estimated 475,000 children aged 0–14 suffer a TBI annually, resulting in over 60,000 hospitalizations and 600,000 emergency department visits [3]. While the immediate neurological consequences of TBI are well-documented, the associated ocular manifestations often receive less attention despite their potential for long-term visual impairment and impact on quality of life.

Ocular involvement in pediatric TBI is not uncommon, with studies indicating that a considerable percentage of children with head injuries experience some form of ocular complication [4]. The anatomical proximity of the eyes to the brain, coupled with the forces involved in traumatic events, places the visual system at high risk. These ocular manifestations can range from subtle visual disturbances to severe, vision threatening conditions, and their timely identification is crucial for appropriate intervention and improved outcomes.

The spectrum of ocular manifestations following pediatric TBI is broad and can include retinal hemorrhages, optic nerve injuries, cranial nerve palsies affecting ocular motility, and various visual problems [5,6]. Beyond direct structural damage, TBI can also lead to functional visual problems such as photophobia, blurred vision, diplopia, and difficulties with visual processing, even in cases of mild TBI [6]. These visual impairments can significantly impact a child’s recovery, rehabilitation, and overall development, affecting their ability to learn, interact, and perform daily activities. Early identification and management of these ocular complications are crucial for optimizing visual outcomes and improving the overall quality of life for pediatric TBI survivors. However, the assessment of ocular health in critically ill pediatric TBI patients in the ICU can be challenging due to their altered mental status, need for sedation, and other life-sustaining interventions. Therefore, a systematic approach to ophthalmological evaluation is essential in this population. This study aims to contribute to the existing literature by providing a detailed prospective analysis of ocular manifestations in pediatric TBI patients admitted to the ICU.

## 2. Methods and Materials

### 2.1. Patients

We prospectively reviewed the medical records of all pediatric patients (≤16 years) diagnosed with traumatic brain injury (TBI) and admitted to the Neurosurgery Intensive Care Unit (ICU) at King Abdullah University Hospital between January 2022 and December 2024.

TBI was defined based on clinical presentation and radiological confirmation (CT or MRI) consistent with brain injury, and according to the U.S. CDC criteria, which include skull fracture, intracranial injury, injury to the optic chiasm, optic pathway, or visual cortex, head injury not otherwise specified, and shaken baby syndrome.

Ocular findings were identified from ophthalmologic consultations, neurosurgical notes, and documented bedside examinations, including manifestations such as visual loss, diplopia, photophobia, ocular motility disorders, corneal defects, and subconjunctival hemorrhage. Young age, sedation, or limited cooperation may affect the reliability of eye examinations; children unable to undergo meaningful ophthalmologic assessment were excluded. Inclusion criteria comprised those pediatric patients under 16 years’ old who sustained a head injury. Patients were excluded if their records lacked sufficient details on ocular assessment, imaging findings, or clinical course. For patients with multiple admissions, data from the initial TBI-related ICU admission were used. This study was approved by the Institutional Review Board of Jordan University of Science and Technology (IRB Approval No.: 36/137/2021, date 14 January 2021). Written informed consent was obtained from patients’ parents.

### 2.2. Data Extraction

Collected data included patient demographics (age, gender), Glasgow Coma Scale (GCS) score on arrival to the emergency department, length of hospital stay, and disposition upon discharge. Clinical information recorded included mode of injury, time from trauma to examination, type and extent of injuries (including neuroimaging), loss of consciousness, and neurological findings.

Ocular injuries were classified according to the type and location of injury. Ophthalmological examinations were performed either in the outpatient or inpatient clinic for ambulatory patients, or at the bedside when necessary.

### 2.3. Perioperative Settings

The examination included best-corrected visual acuity (BCVA), pupillary response, extraorbital injury assessment, extraocular motility, anterior segment evaluation with a slit lamp, visual field assessment by confrontation or perimetry, intraocular pressure measurement with applanation tonometry, and dilated fundus examination. Additional investigations such as CT, MRI, and B-scan ultrasonography were performed when indicated. Patients were followed up at 1 week, 4 weeks, and 24 weeks’ post-injury.

All patients underwent comprehensive ophthalmic examinations. The BCVA was converted to LogMAR visual acuity. Goldmann applanation tonometer was utilized to measure the intraocular pressure. Slit lamp and indirect biomicroscopes were used to assess the lens status, anterior segment, and fundus conditions. The appropriate gonio-lens were utilized to assess the angle structures. Humphrey automated perimetry was applied for most patients to investigate and follow the visual fields of the patients. Additional tests such as OCT imaging, visual-evoked potentials (VEP), and orthoptic assessment of extraocular muscle function were not performed.

Our protocol for the initial assessment and management of children with TBI, includes obtaining a brain CT scan, and additional imaging of the cervical spine. If the patient has impending clinical signs of brain herniation (pupillary abnormalities, hemodynamic alternations suggestive of Cushing’s triad, and extensor posturing) or CT scan findings of large mass lesion (subdural hemorrhage, epidural hemorrhage, intracerebral hemorrhage, massive edema with midline shifting, or herniation), immediate surgery for decompressive craniectomy and evacuation of the mass lesion was performed. While if there are no signs of brain herniation (clinically or radiologically), the patient then admitted to the pediatric neurointensive care unit for further treatment.

Initial treatment includes endotracheal intubation, sedation using fentanyl or propofol, mechanical ventilation to keep PaCO_2_ between 35 and 40 mmHg, and it is not recommended to induce hyperventilation so that PaCO_2_ is lower than 30 mmHg during the first 48 h after TBI. However, if hyperventilation is needed to treat refractory intracranial hypertension, it is suggested that neuromonitoring is used to detect cerebral ischemia, head of bed elevation to 30–45°, brief hyperventilation targeting a PaCO_2_ of 30 mmHg, administration of 0.5 g/kg mannitol or hypertonic saline (1–3 mL/kg up to a maximum of 250 mL for 3% saline), prophylactic antiepileptic medications to prevent early post traumatic seizures using either phenytoin (loading dose of 15 mg/kg, then maintenance dose of 50 mg/kg/day divided over 3 doses).

Postoperative ICU management continues with sedation, ventilation, and administration of hypertonic saline or mannitol. If hyperosmolar therapy and propofol infusion (titrated to deep sedation) does not lower ICP, then we consider putting the patient into barbiturate coma.

### 2.4. Statistical Analysis

Data were entered into a spreadsheet and analyzed using IBM SPSS Statistics for Windows, Version 26.0 (Armonk, NY, USA). Nominal variables were expressed as frequency and percentage, while continuous variables were reported as mean ± standard error of the mean (SEM). Comparisons between groups were performed using the Chi-square test for categorical variables and the Student’s *t*-test for continuous variables. A *p*-value ≤ 0.05 was considered statistically significant. Multivariable logistic regression with penalization and bootstrap resampling was performed to estimate adjusted odds ratios (aOR) with 95% confidence intervals for prespecified predictors.

## 3. Results

### 3.1. Patient Demographics

A total of 38 pediatric patients with traumatic brain injury (TBI) admitted to the intensive care unit (ICU) were included in this study. The median age of the entire cohort was 8 years. Males comprised 55.3% (n = 21) of the population. Out of the total, 20 patients (52.6%) presented with ocular findings, while 18 patients (47.4%) did not.

The median age of patients with ocular findings was 10 years, significantly higher than 6 years in those without ocular findings (*p* = 0.007). The median Glasgow Coma Scale (GCS) score on admission was significantly lower in the ocular group (11) compared to the non-ocular group (14) (*p* = 0.016), indicating more severe neurological impairment among those with ocular involvement. A greater proportion of males was observed among patients with ocular findings (75.0%) compared to those without (33.3%) (*p* = 0.030) (Table 1). Continuous variables (age, GCS) were compared using the Mann-Whitney U test, and categorical variables were assessed using the Chi-square or Fisher’s exact test, as appropriate.

### 3.2. Ocular Findings

Surgical intervention was performed significantly more frequently in patients with ocular findings compared to those without ocular findings (60.0% vs. 22.2%, *p* = 0.025). Orbital fractures were also significantly more common in the ocular group (40.0% vs. 5.6%, *p* = 0.021). Extraocular muscle (EOM) limitation, corneal defects, punctate epithelial erosions (PEEs), subconjunctival hemorrhage, and chemosis were each observed at significantly higher rates in the ocular group (*p* = 0.048 for all). Signs suggestive of basal skull fracture (e.g., raccoon eyes, ecchymosis) were significantly associated with ocular findings (*p* = 0.036). The site of head injury differed significantly between groups, with craniofacial trauma more frequently observed in the ocular group (*p* = 0.027). Additionally, male gender was more prevalent among patients with ocular findings (*p* = 0.030).

No statistically significant associations were identified between ocular findings and papilledema, Roth spots, relative afferent pupillary defect (RAPD), or mechanism of injury (*p* > 0.05 for all). (Table 2) Fisher’s exact test was applied for analyses in which expected cell counts were less than five. In the multivariable logistic regression model, orbital fracture was the only independent predictor of ocular findings (aOR 2.22, 95% CI 1.17–3.57, *p* = 0.02). Age, sex, GCS, surgical intervention, and signs of basal skull fracture were not significantly associated after adjustment. No cases of delayed complications were reported.

## 4. Discussion

This prospective analysis of 38 pediatric patients with traumatic brain injury (TBI) admitted to the intensive care unit provides valuable insights into the prevalence and characteristics of ocular manifestations in this vulnerable population. Our findings demonstrate that ocular complications occur in 52.6% of pediatric TBI patients requiring ICU admission, which is substantially higher than previously reported rates in general pediatric TBI populations [7,8]. This elevated prevalence likely reflects the severity of injuries in our ICU cohort, as evidenced by the significantly lower Glasgow Coma Scale scores in patients with ocular findings compared to those without (*p* = 0.016).

The 52.6% prevalence of ocular manifestations in our study aligns with the upper range of previously reported rates in pediatric TBI populations. Gise et al. [1] analyzed the National Trauma Data Bank and found that among 58,765 pediatric patients admitted for trauma with ocular injuries, 32,173 (54.7%) were diagnosed with TBI, suggesting a strong association between these conditions. Similarly, Shokunbi et al. [4] reported ocular complications in 28% of children with head injury, though their study included a broader spectrum of TBI severity. The higher prevalence in our study may be attributed to the exclusive focus on ICU patients, who by definition have more severe injuries requiring intensive monitoring and care.

Our finding that patients with ocular manifestations were significantly older (median age 10 years vs. 6 years, *p* = 0.007) is consistent with previous literature suggesting that older children may be at higher risk for certain types of ocular complications following TBI [5]. This age-related difference may reflect developmental changes in skull anatomy, with older children having more developed facial bones that can transmit forces to the orbital structures during trauma [6]. Additionally, older children are more likely to engage in high-risk activities such as sports and motor vehicle-related activities, which can result in more severe craniofacial trauma [1,9]. Regarding gender difference, male predominance in our ocular manifestation group is consistent with epidemiological data showing that males are at higher risk for both TBI and ocular trauma [7]. This gender disparity likely reflects behavioral differences, with males more frequently engaging in high-risk activities and contact sports that predispose to traumatic injuries [10].

The significant association between lower GCS scores and the presence of ocular manifestations (*p* = 0.016) underscores the relationship between injury severity and ocular complications. This finding is supported by previous research demonstrating that more severe TBI is associated with a higher likelihood of ocular involvement [11]. The anatomical proximity of the visual system to critical brain structures means that forces sufficient to cause severe TBI often simultaneously affect the visual pathways, extraocular muscles, and orbital structures [12].

The higher rate of surgical intervention in patients with ocular findings (60.0% vs. 22.2%, *p* = 0.025) further supports the association between injury severity and ocular manifestations. Patients requiring neurosurgical intervention typically have more severe intracranial pathology, which may be accompanied by greater forces of impact that can damage orbital and ocular structures [13]. This finding has important clinical implications, as it suggests that patients undergoing neurosurgical procedures should receive comprehensive ophthalmological evaluation to identify and manage concurrent ocular injuries.

Regarding orbital bone fracture and ocular findings, the significantly higher prevalence of orbital fractures in patients with ocular findings (40.0% vs. 5.6%, *p* = 0.021) highlights the importance of craniofacial trauma in the pathogenesis of ocular complications following pediatric TBI. Orbital fractures can result from direct impact to the face or from transmitted forces through the skull base [14]. These fractures can lead to various complications including diplopia, enophthalmos, and restriction of extraocular movement, all of which can significantly impact visual function and quality of life [15]. Importantly, our multivariable analysis confirmed orbital fracture as the only independent predictor of ocular manifestations.

Given these mechanisms, it is not surprising that fractures involving the skull base may also contribute to ocular injury through similar pathways, particularly when neurovascular structures related to vision are affected. The association between signs of basal skull fracture (such as raccoon eyes and ecchymosis) and ocular findings (*p* = 0.036) is particularly noteworthy. Basal skull fractures can damage cranial nerves involved in vision and ocular motility, including the optic nerve (cranial nerve II), oculomotor nerve (cranial nerve III), trochlear nerve (cranial nerve IV), and abducens nerve (cranial nerve VI) [16]. The presence of periorbital ecchymosis (raccoon eyes) specifically indicates bleeding from fractures of the anterior cranial fossa, which can extend into the orbital cavity and affect ocular structures. This potential for cranial nerve injury provides a plausible link to the extraocular muscle limitations observed in our study. The significant EOM limitation and ocular findings in our study (*p* = 0.048) reflects the vulnerability of the ocular motor system to traumatic injury. EOM dysfunction can result from several mechanisms including direct muscle injury, entrapment due to orbital fractures, cranial nerve palsies, or brainstem injury affecting supranuclear control of eye movements [17]. In pediatric patients, EOM dysfunction is particularly concerning as it can lead to amblyopia and permanent visual impairment if not promptly identified and treated [18].

The development of diplopia following pediatric TBI can have profound implications for a child’s development, affecting their ability to perform academic tasks, participate in sports, and navigate their environment safely. Early identification and management of EOM dysfunction through orthoptic assessment and appropriate interventions, including prism glasses or surgical correction, are crucial for optimizing visual outcomes [19].

The long-term consequences of visual impairment in children extend far beyond mere visual acuity, impacting multiple facets of neurodevelopment, academic performance, social integration, and overall quality of life. Visual input is fundamental to a child’s developing brain, influencing spatial awareness, motor skills, and cognitive functions. Early visual deficits can disrupt critical periods of neurodevelopment, potentially leading to permanent functional limitations [20]. In an academic setting, visual impairment can severely hinder learning, reading, and participation in classroom activities, contributing to educational underachievement and increased risk of school dropout [21]. Socially, children with visual impairments may face challenges in peer interaction, developing social cues, and participating in recreational activities, leading to isolation and reduced quality of life [22]. Therefore, the high prevalence of ocular manifestations in pediatric TBI, as observed in our study, underscores a significant public health concern.

Our study was limited by the absence of advanced diagnostic modalities such as Visual Evoked Potentials, Optical Coherence Tomography, and detailed retinal imaging. This limitation is often a reflection of the practical challenges and resource constraints prevalent in an intensive care unit setting. However, the value of these advanced modalities in pediatric TBI cannot be overstated. VEP can objectively assess the integrity of the visual pathways, even in non-verbal or uncooperative patients, providing crucial information about optic nerve function and cortical visual processing [23]. OCT offers high-resolution cross-sectional imaging of the retina and optic nerve head, enabling the detection of subtle structural changes that may not be apparent on clinical examination [24,25]. Retinal imaging, including wide-field fundus photography, can document and monitor retinal hemorrhages, which are particularly relevant in cases of abusive head trauma but can also occur in accidental TBI [26]. Studies have demonstrated the feasibility of portable OCT systems in pediatric populations, even in challenging environments, highlighting their potential utility [27].

Our study has other limitations that warrant consideration. First, we relied on existing medical records, which may have led to incomplete documentation of all ocular findings or variations in the thoroughness of ophthalmological examinations. Second, the sample size of 38 patients, while providing valuable insights, limits the generalizability of our findings to the broader pediatric TBI population. Third, baseline ophthalmologic records were not available as traumatic brain injuries occur acutely and unexpectedly. Future prospective studies with larger cohorts and standardized ophthalmological assessments are needed to confirm these associations and explore additional ocular manifestations.

Furthermore, our study focused on patients admitted to the ICU, which represents a more severely injured subset of pediatric TBI patients. Future research could investigate ocular manifestations across the full spectrum of TBI severity, including mild TBI, to better understand the overall burden of visual impairment in this population

## 5. Conclusions

In conclusion, our study highlights the high prevalence of ocular manifestations in pediatric TBI patients admitted to the ICU, emphasizing the need for routine and comprehensive ophthalmological evaluation in this population. The significant associations between ocular findings and injury severity, male gender, older age, and specific types of craniofacial trauma underscore the importance of a multidisciplinary approach to managing these complex cases. Early identification and appropriate management of ocular complications are critical for optimizing visual outcomes and improving the overall quality of life for pediatric TBI survivors.

## Figures and Tables

**Table 1:** Demographic and Clinical Characteristics of Included Patients.

Variables	N (%)			*p* Value
	Total N = 38	No Ocular Findings N = 18	Ocular Findings N = 20	
**Age**				0.007
Median	8	6	10	
**GCS on Admission**				0.016
Median	11.5	14	11	
**Male**				0.030
N (%)	21 (55.3%)	6 (33.3%)	15 (75.0%)	
**Surgery Performed**				0.025
N (%)	16 (42.1%)	4 (22.2%)	12 (60.0%)	
**Site of Head injury**	Mostly cranial	Mostly cranial	Mostly craniofacial	0.027

**Table 2:** Ocular Findings and Their Association with Clinical Features.

Variables	N (%)			*p* Value
	Total N = 38	No Ocular Findings N = 18	Ocular Findings N = 20	
**Feature**				
Eyelid Swelling (%)	28.9	0	55.0	<0.001
Orbital Fracture (%)	34.2	5.6	40.0	0.021
EOM Limitation (%)	10.5	0	25.0	0.048
Corneal Defect (%)	7.9	0	25.0	0.048
PEEs (%)	N/A	0	25.0	0.048
Subconjunctival hemorrhage (%)	13.2	0	25.0	0.048
Chemosis (%)	10.5	0	25.0	0.048
Signs of Basal Skull Fracture	52.6	Fewer	More	0.036
Papilledema, RAPD, Roth spots	15.8	Not significant	Not significant	>0.05

## Data Availability

The original contributions presented in this study are included in the article. Further inquiries can be directed to the corresponding authors.
